# An Integrated Approach Utilizing Single-Cell and Bulk RNA-Sequencing for the Identification of a Mitophagy-Associated Genes Signature: Implications for Prognostication and Therapeutic Stratification in Prostate Cancer

**DOI:** 10.3390/biomedicines13020311

**Published:** 2025-01-27

**Authors:** Yuke Zhang, Li Ding, Zhijin Zhang, Liliang Shen, Yadong Guo, Wentao Zhang, Yang Yu, Zhuoran Gu, Ji Liu, Aimaitiaji Kadier, Jiang Geng, Shiyu Mao, Xudong Yao

**Affiliations:** 1 Department of Urology, Shanghai Tenth People’s Hospital, School of Medicine, Tongji University, 301 Middle Yan Chang Road, Shanghai 200072, China; yukezhang001@gmail.com (Y.Z.); ding0710@foxmail.com (L.D.); 2231200@tongji.edu.cn (Z.Z.); 1731191@tongji.edu.cn (Y.G.); zhangwentao98@163.com (W.Z.); sdykdxyy@163.com (Y.Y.); guzhuorango@126.com (Z.G.); drliu7087797@163.com (J.L.); 1911637@tongji.edu.cn (A.K.); gengjiangsn@sina.com (J.G.); 2 Urologic Cancer Institute, School of Medicine, Tongji University, Shanghai 200072, China; 3 Department of Urology, Ningbo Yinzhou People’s Hospital, 251 Baizhang East Road, Ningbo 315100, China; shenliliang@sina.com

**Keywords:** prostate cancer, single-cell RNA sequencing, prognosis, biomarker, mitophagy-related genes

## Abstract

**Introduction**: Prostate cancer, notably prostate adenocarcinoma (PARD), has high incidence and mortality rates. Although typically resistant to immunotherapy, recent studies have found immune targets for prostate cancer. Stratifying patients by molecular subtypes may identify those who could benefit from immunotherapy. **Methods**: We used single-cell and bulk RNA sequencing data from GEO and TCGA databases. We characterized the tumor microenvironment at the single-cell level, analyzing cell interactions and identifying fibroblasts linked to mitophagy. Target genes were narrowed down at the bulk transcriptome level to construct a PARD prognosis prediction nomogram. Unsupervised consensus clustering classified PARD into subtypes, analyzing differences in clinical features, immune infiltration, and immunotherapy. Furthermore, the cellular functions of the genes of interest were verified in vitro. **Results**: We identified ten cell types and 160 mitophagy-related single-cell differentially expressed genes (MR-scDEGs). Strong interactions were observed between fibroblasts, endothelial cells, CD8^+^ T cells, and NK cells. Fibroblasts linked to mitophagy were divided into six subtypes. Intersection of DEGs from three bulk datasets with MR-scDEGs identified 26 key genes clustered into two subgroups. COX regression analysis identified seven prognostic key genes, enabling a prognostic nomogram model. High and low-risk groups showed significant differences in clinical features, immune infiltration, immunotherapy, and drug sensitivity. In prostate cancer cell lines, CAV1, PALLD, and ITGB8 are upregulated, while CLDN7 is downregulated. Knockdown of PALLD significantly inhibits the proliferation and colony-forming ability of PC3 and DU145 cells, suggesting the important roles of this gene in prostate cancer progression. **Conclusions**: This study analyzed mitophagy-related genes in PARD, predicting prognosis and aiding in subtype identification and immunotherapy response analysis. This approach offers new strategies for treating prostate cancer with specific molecular subtypes and helps develop potential biomarkers for personalized medicine strategies.

## 1. Introduction

Prostate cancer is one of the most common and deadliest malignancies worldwide. Although comprehensive treatments based on androgen deprivation therapy and chemotherapy have significantly improved patient prognosis in recent years, once the disease progresses to the castration-resistant stage, the prognosis for patients becomes very poor. Prostate adenocarcinoma (PARD) is the predominant form of prostate cancer, prevalent among men and associated with significant morbidity and mortality [1]. Clinically, the pharmacological treatments for inoperable PARD mainly include androgen deprivation therapy, novel hormone agents represented by androgen receptor signaling inhibitors, and paclitaxel-based chemotherapy [2,3,4]. Although these drugs improve patient prognosis, most patients develop drug resistance, progress to castration-resistant PARD, and have a poor prognosis [5,6]. Immunotherapy shows promise in cancer treatment, yet only a minority of PARD patients benefit from immune checkpoint inhibitors [7,8]. Hence, identifying appropriate biomarkers to predict immunotherapy response in PARD patients is crucial. Doing so could enhance patient prognosis and quality of survival, aligning with the objective of precision medicine. The tumor microenvironment (TME) is pivotal in tumor progression and invasion, significantly influencing the efficacy of immunotherapy and, consequently, patient survival. An in-depth analysis of TME could assist in identifying biomarkers relevant to immunotherapy.

Mitochondria play a crucial role in cellular metabolism as the powerhouse of the cell [9]. Under damaged or specific developmental conditions, mitochondria undergo selective elimination via a process known as mitophagy [9]. Mitophagy serves to eliminate aged or damaged mitochondria, thereby preserving the integrity of the mitochondrial pool and ensuring cellular homeostasis [10], but it has also been found to play a role in promoting or limiting tumorigenesis, and failure of mitophagy facilitates tumorigenic stress and tumorigenesis [11], and playing a dual role in chemotherapy, radiotherapy, and immunotherapy [12].

The advancement of single-cell RNA sequencing (scRNA-seq) technology enables the amplification and sequencing of the entire genome at the single-cell level. This capability facilitates the exploration of cell abundance and functional status within the TME and allows for the analysis of molecular characteristics within specific subpopulations [13]. In contrast to traditional bulk transcriptome analysis, scRNA-seq technology can fully unveil the heterogeneity among tumor cells and the intricate complexity of the TME. In recent years, this technique has been widely used in the field of PARD [14,15]. However, the distribution of the expression of mitophagy-related genes (MRGs) in PARD and the heterogeneity among different cell types have not been investigated; meanwhile, whether MRGs could be used for the prognostic assessment of PARD patients and the prediction of the response to immune checkpoint inhibitor therapy is not yet known. Therefore, in this study, we analyzed the significance of MRGs in PARD development at the single-cell RNA level, identified prognostically relevant signatures and constructed a prognostic model. Furthermore, we elucidated the correlation between MRGs and the tumor immune microenvironment, genomic heterogeneity, therapeutic response, and the significance of drug selection in PARD. These discoveries hold promise in furnishing dependable biomarkers and therapeutic approaches for the future clinical management of PARD.

## 2. Materials and Methods

### 2.1. Data Collection and Processing

Single-cell RNA sequencing data, GSE185344, were downloaded from the Gene Expression Omnibus (GEO, https://www.ncbi.nlm.nih.gov/geo/, accessed on 14 March 2024). The dataset was sequenced on a GPL24676 (Illumina NovaSeq 6000, San Diego, CA, USA) using 10× genomics technology, and the species source is Homo sapiens. The dataset included 7 prostate cancer tissue samples, and 7 matched normal prostate tissue samples from prostate cancer patients, all of which were included in this study.

We obtained bulk RNA-sequencing data and clinical information of PRAD patients from the Cancer Genome Atlas (TCGA) database (https://portal.gdc.cancer.gov, accessed on 14 March 2024). Additionally, datasets GSE46602, GSE69223, and GSE38241 were downloaded from the GEO database to validate model predictions (Table S1). The bulk RNA-sequencing data underwent log2 transformation during processing. Samples without survival data were excluded. Microsatellite instability (MSI) and tumor mutational burden (TMB) data were obtained from cBioPortal for Cancer Genomics (http://cbioportal.org, accessed on 14 March 2024). A total of 4660 MRGs were obtained from the GeneCards database using “Mitophagy” and “Protein Coding” as filtering criteria (Table S2).

### 2.2. Analysis of scRNA-Seq Data

To perform principal component analysis (PCA), the normalized scRNA-seq data was first scaled using the ScaleData function in Seurat to ensure that each gene had a mean of 0 and a standard deviation of 1. This step removes any potential biases due to differences in gene expression scales. We then applied PCA to the top 3000 highly variable genes identified using the FindVariableFeatures function in the Seurat package (3.6.2), which were selected based on their variance across the cells. For PCA, we used the RunPCA function in Seurat with the top 3000 variable genes as input. We retained the first 40 principal components (PCs) based on the variance explained, as determined by examining the “elbow” plot. The number of components was chosen by inspecting the scree plot and selecting the PCs that contributed to the majority of the variance in the dataset. The resulting PCA coordinates were visualized using the VizDimLoadings function to assess which genes contributed most to each principal component. To determine the appropriate number of principal components for downstream analysis, we used the ElbowPlot function, which helps identify the point where the variance explained by additional components begins to level off. Unsupervised clustering and visualization were carried out using uniform manifold approximation and projection (UMAP) with a resolution of 0.8. The FindAllMarkers function was utilized to compare differentially expressed genes (DEGs) between clusters. Cell subtypes within different clusters were annotated using the SingleR package (3.6.2). Cell-cell communication analysis and network visualization were conducted using the Python packages CellPhoneDB (1.1.0), and genes of interest were scored using the AUCell algorithm. Trajectory analysis was performed using the monocle2 package (2.4.0).

### 2.3. Enrichment Analysis and Consensus Clustering Analysis

Gene ontology (GO) pathway analyses were performed using the ClusterProfiler package (4.3.3). The *p*-values were adjusted using the Benjamini-Hochberg method. Unsupervised cluster analysis was conducted using the consensus clustering method implemented in the ConsensusClusterPlus package. The optimal number of clusters was determined by assessing the cumulative distribution function (CDF) curve and the CDF delta area curve.

### 2.4. Prediction Model Construction

To build a prediction nomogram model, we conducted univariate and multivariate Cox regression analyses on relevant genes. Genes with a *p*-value < 0.1 in the univariate regression analyses were included in the multivariate regression analyses to construct the model using the rms package. Model reliability was evaluated using receiver operating characteristic (ROC) curves, calibration curves, and decision curve analysis (DCA) curves.

### 2.5. Evaluation of Tumor Immune Microenvironment

We calculated the infiltration scores of 29 immune cells or pathways obtained from previous studies using the ssGSEA algorithm from the “GSVA” package. The Mann-Whitney test was then employed to assess the significance of these scores. The ESTIMATE algorithm was used to assess immune scores, stromal scores, and ESTIMATE scores based on expression data. The CIBERSORT algorithm was employed to evaluate 22 types of immune cell infiltration levels.

### 2.6. Drug Sensitivity Analysis

We obtained the half-maximal inhibitory concentration (IC50) values from the Genomics of Drug Sensitivity in Cancer (GDSC) database (https://www.cancerrxgene.org/, accessed on 14 May 2024); the efficacy of common anticancer drugs between subgroups was calculated using the pRRophetic algorithm.

### 2.7. Cell Culture and Transfection

PC3 and DU145 human prostate cancer cell lines, along with LNCaP and 22RV-1 cell lines, were cultured in RPMI-1640 medium supplemented with 10% fetal bovine serum (FBS), penicillin (100 U/mL), and streptomycin (100 μg/mL) at 37 °C in a 5% CO_2_ incubator. The normal human prostate epithelial cell line RWPE-1 was cultured in a Defined Keratinocyte SFM (1X) medium (Gibco, Grand Island, NY, USA). PC3 and DU145 cells were transfected with PALLD-targeting siRNAs (PALLD-si#1, PALLD-si#2) or non-targeting control siRNA (siNC) using Lipofectamine RNAiMAX (Invitrogen, Carlsbad, CA, USA), following the manufacturer’s instructions.

### 2.8. RNA Interference Efficiency

PALLD knockdown efficiency was validated using quantitative PCR (qPCR). Total RNA was extracted using TRIzol (Thermo Fisher or Tiangen, Beijing, China). About 2 μg of RNA was reverse transcribed into cDNA using SuperScript IV (Thermo Fisher) or a cDNA synthesis kit (#EP0441; Thermo Fisher Scientific, Waltham, MA, USA). qPCR was performed using SYBR Green Master Mix (Bio-Rad, Hercules, CA, USA) or PerfectStart Green qPCR SuperMix (#AQ601-04, Transgen, Beijing, China). GAPDH was used as the internal control, and relative mRNA expression was calculated using the 2^−ΔΔCt^ method. The primer sequences for GAPDH and 7 additional genes (CAV1, CLDN7, ITGB8, PALLD, PDLIM5, TACC1, and TGFBR3) are listed in Table S3.

### 2.9. Cell Proliferation Assay

Cell proliferation was assessed using the CCK8 assay. Cells were seeded in 96-well plates and treated with siRNA for 24 h. CCK8 was added at days 1–5, and absorbance at 450 nm was measured to evaluate proliferation rates.

### 2.10. Colony Formation Assay

Cells were seeded in 6-well plates, transfected with siRNA, and cultured for 10–14 days. Colonies were stained with crystal violet and counted under a microscope.

### 2.11. Statistical Analysis

All statistical analyses were conducted using R software version 4.12 (http://www.R-project.org, accessed on 22 August 2024). Student’s *t*-test or Wilcoxon’s rank sum test was employed to detect significant differences between two independent groups, with *p* < 0.05 considered statistically significant.

## 3. Results

### 3.1. Identification of Mitophagy-Related Differentially Expressed Genes

**Figure 1:**
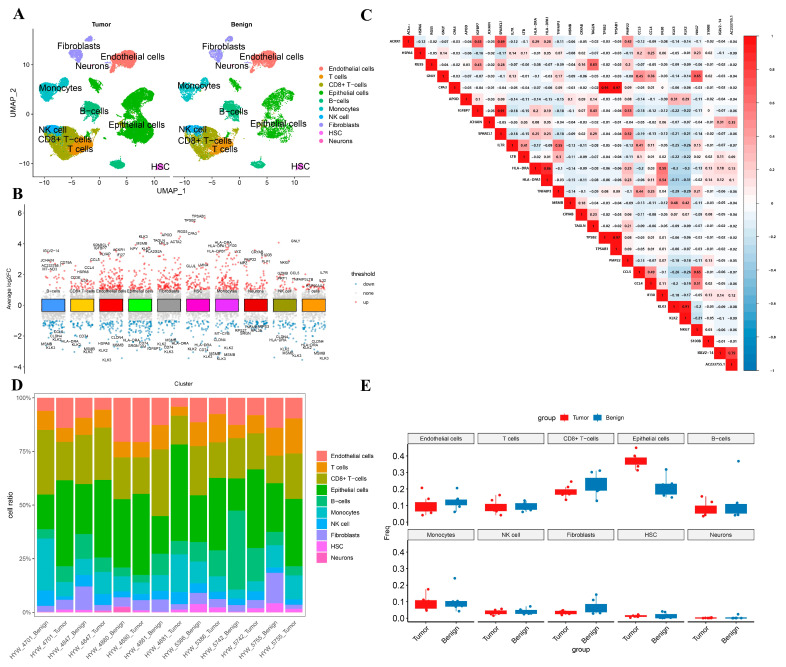
(**A**) Single-cell RNA landscape of the microenvironment in prostate cancer and benign control group; (**B**) Volcano plot of top5 up-and down-regulated genes for cell types; (**C**) Correlation analysis of top3 upregulated genes in cell types; (**D**) Proportion of cell types in different samples; (**E**) Differences in the proportion of different cell types between the tumor and benign groups.

The flow chart of this study is shown in Figure S1. The scRNA-seq data used in this study includes 38,232 cells after quality control. Figure S2A displays the range of gene numbers detected, the sequencing depth, and the percentage of mitochondrial content in each group. After normalizing the data, we selected the top 3000 highly variable genes for further analysis (Figure S2B). PCA was employed for initial dimensionality reduction, and the top 40 principal components were selected for further analysis. Subsequently, we applied UMAP for dimensionality reduction visualization, classifying all cells into 27 clusters (Figure S2C). Using the SingleR package (2.6.0), we identified 10 cell types (Figure 1A). Specifically, clusters 4 and 13 were annotated as B cells, clusters 2, 7, 15, 19, 25, and 26 as CD8^+^ T cells, clusters 0 and 21 as Endothelial cells, clusters 3, 5, 8, 9, 11, 12, 16, 22, and 23 as Epithelial cells, clusters 14 and 20 as Fibroblasts, cluster 18 as hematopoietic stem cells, clusters 6 and 17 as Monocytes, cluster 24 as Neurons, cluster 10 as NK cells, and cluster 1 as T cells (non-CD8^+^) (Figure S2C and Figure 1A).

For the ten annotated cell types, we calculate DEGs between types, setting the threshold at |logFC| > 1 and P.adj < 0.05. The top five upregulated and downregulated genes for each cell type were displayed (Figure 1B). Using the Pearson correlation analysis algorithm, we analyzed the correlation among these 30 DEGs. The results showed a predominantly negative correlation among most DEGs, suggesting that these DEGs might share a similar expression pattern in PRAD patients (Figure 1C). Additionally, the proportion of various cell types varied greatly among different samples, reflecting the significant heterogeneity of PRAD (Figure 1D). Compared to the normal control group, the proportion of epithelial cells and monocytes increased in the PARD group, whereas the proportions of endothelial cells, CD8^+^ T cells, and fibroblasts decreased (Figure 1E). We intersected the upregulated DEGs with MRGs, defining 160 genes as mitophagy-related single-cell differently expressed genes (MR-scDEGs) (Table S4).

### 3.2. Cell Communication Analysis and AUCell Score of MR-scDEGs

**Figure 2:**
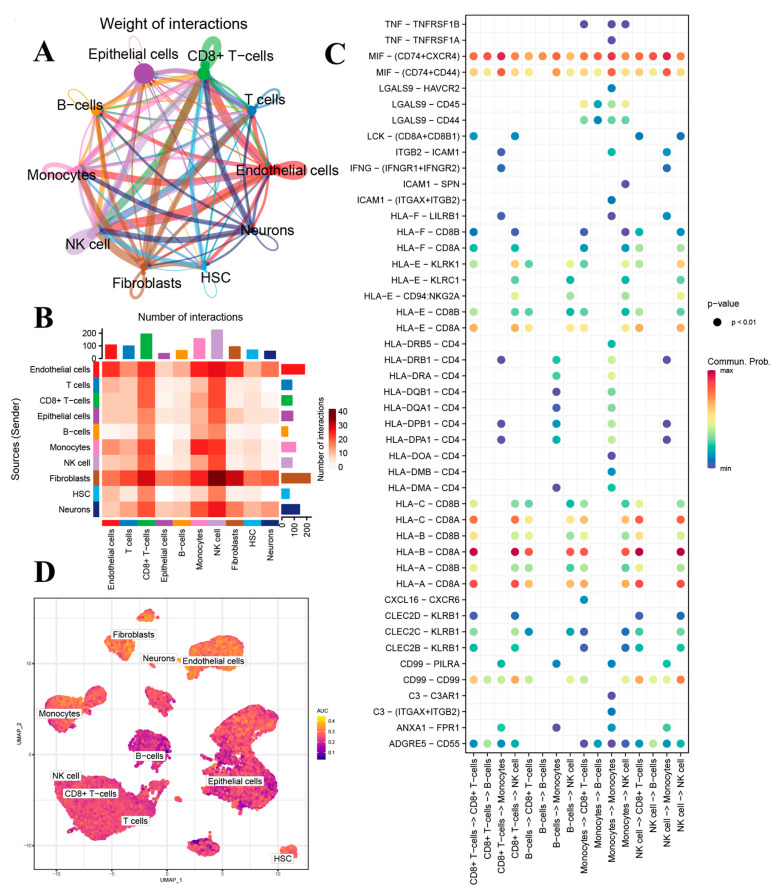
Cell-cell communication displaying interaction weight (**A**) and interaction number (**B**); (**C**) Receptor-ligand interaction networks between different cell types; (**D**) Automatic cell identification and clustering score of different cell types based on mitophagy-related single-cell differently expressed genes.

The weight and number of interactions between different cell types are illustrated in Figure 2A,B. Notably, the interactions between fibroblasts and endothelial cells with CD8+ T cells and NK cells were particularly strong. Subsequently, we visualized the interaction strengths of ligands across different cell subtypes (Figure 2C). We observed that certain ligands, such as MIF—(CD74+CXCR4) and HLA-B—CD8A, exhibited higher positive correlation strengths among fibroblasts, endothelial cells, CD8^+^ T cells, and NK cells. In contrast, ligands like TNF—TNFRSF1B and HLA-F—CD8B showed higher negative correlation strengths. Additionally, we employed the AUCell package to demonstrate the activity levels of MR-scDEGs across different cell types. The results indicated that fibroblasts, endothelial cells, and neurons had higher AUCell scores (Figure 2D). After comprehensive consideration, fibroblasts were further analyzed as a subgroup of interest.

### 3.3. Subtype Analysis of Fibroblasts

**Figure 3:**
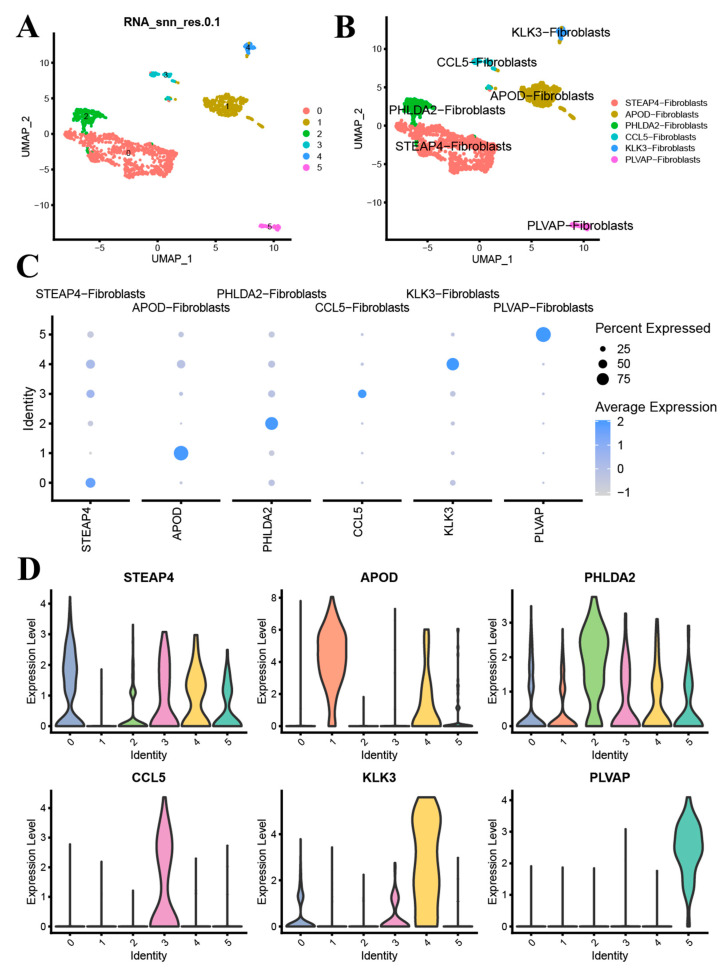
Fibroblasts were divided into six subtypes at 0.1 resolution (**A**) and annotated based on marker genes (**B**), Bubble (**C**) and violin plots (**D**) of marker genes.

To explore new subtypes of fibroblasts, we conducted a separate re-clustering, setting the resolution to 0.1, and categorized the fibroblasts into six clusters (Figure 3A). By analyzing the DEGs between clusters, these were annotated as STEAP4-fibroblasts, APOD-fibroblasts, PHLDA2-fibroblasts, CCL5-fibroblasts, KLK3-fibroblasts, and PLVAP-fibroblasts (Figure 3B). We visualized their marker genes using a bubble plot and violin plot (Figure 3C,D).

We further analyzed the differentiation dynamics of different fibroblast subtype populations. As pseudotime progressed, the abundance of APOD-Fibroblasts gradually decreased, while STEAP4-Fibroblasts, PHLDA2-Fibroblasts, and CCL5-Fibroblasts increased. However, no significant changes were observed in the abundance of KLK3-Fibroblasts and PLVAP-Fibroblasts over pseudotime (Figure S3A–D). Additionally, we visualized the top 20 DEGs with the highest and lowest logFC values (Figure S3E).

### 3.4. Metabolism-Related Key Genes Identification and Analysis

**Figure 4:**
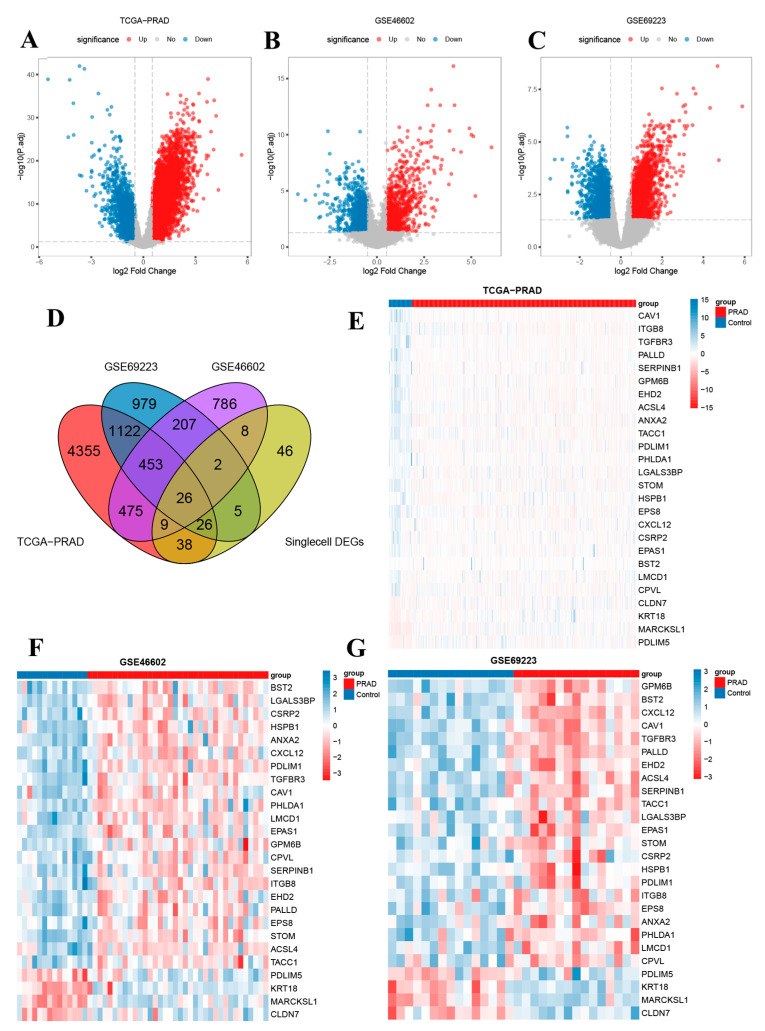
Volcano plot of differently expressed genes between different groups in TCGA-PRAD (**A**), GSE46602 (**B**) and GSE69223 dataset (**C**); (**D**) Identification of 26 key genes in Venn diagram; (**E**–**G**) Heatmap of 26 key genes between different groups in TCGA-PRAD (**E**), GSE46602 (**F**) and GSE69223 dataset (**G**).

DEGs analysis was conducted between tumor and control groups in the TCGA-PRAD (Figure 4A), GSE46602 (Figure 4B), and GSE69223 datasets (Figure 4C), with |logFC| > 0.5 and P.adj < 0.05. The intersection of DEGs from these three datasets with MR-scDEGs resulted in 26 genes defined as key genes (Figure 4D). Heatmaps were used to visualize the expression patterns of these key genes across three datasets (Figure 4E–G). GO enrichment analysis of the key genes revealed enrichment primarily in BP, such as response to hypoxia, response to decreased oxygen levels, response to oxygen levels, regulation of actin filament-based process, and organic hydroxy compound transport. They were also enriched in cell components (CC), including membrane raft, focal adhesion, sarcomere, cell-cell junction, and primary lysosome. Additionally, enrichment was observed in molecular function (MF), such as cell-cell adhesion mediator activity, cell adhesion mediator activity, actin binding, cadherin binding involved in cell-cell adhesion, and enzyme inhibitor activity. The results of the GO functional enrichment analysis were presented using bar charts (Figure S4A). Furthermore, the enriched BP pathways (Figure S4B), CC pathways (Figure S4C), and MF pathways (Figure S4D) from the GO gene function enrichment analysis were visualized in the form of network diagrams.

### 3.5. Identification and Differential Analysis of Subtypes of Prostate Cancer

**Figure 5:**
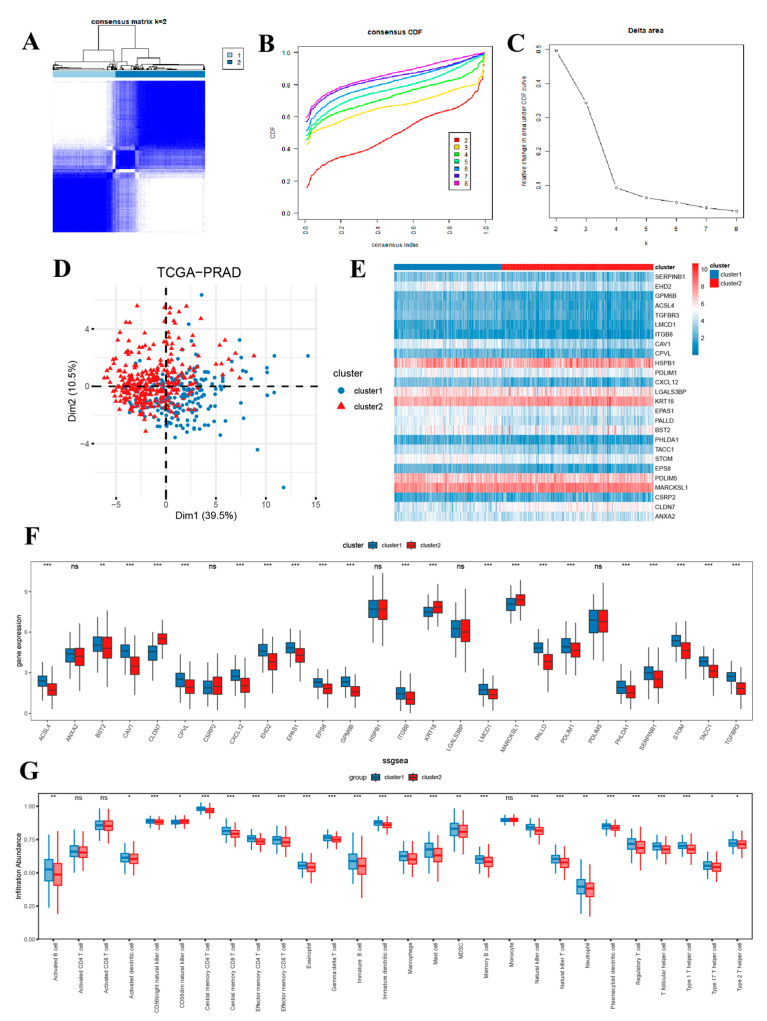
(**A**) Consensus clustering matrix showing the clustering agreement between samples when k (number of clusters) = 2; (**B**) Representative cumulative distribution function curve showing the clustering results for k ranging from 2 to 8; (**C**) Relative changes in cumulative distribution function delta area curves, which measure the stability of clustering across different values of k; (**D**) Principal component analysis of two groups; Heatmap (**E**) and box plot (**F**) of 26 key genes expressed in two clusters; (**G**) Single-sample gene-set enrichment analysis in two clusters. * *p* < 0.05, ** *p* < 0.01, *** *p* < 0.001; ns, non significance.

Unsupervised clustering was performed based on 26 key genes, and all samples were clustered into two subtypes, cluster1 and cluster2 (Figure 5A). The optimal segmentation efficiency was achieved at k = 2 by comprehensive analysis of the CDF curves (Figure 5B) and delta area (Figure 5C). The PCA clustering results (Figure 5D) and the heatmap (Figure 5E) also showed significant differences between the two isoforms. Figure 5F demonstrated the difference in the expression of 26 key genes between the two isoforms, with the vast majority of key genes significantly upregulated in cluster1 relative to cluster2, while CLDN-7, KRT18, and MARCKSL1 were significantly down-regulated in cluster1 relative to cluster2. The ssGSEA algorithm was further used to explore the differences in immune infiltration between the two subtypes, comparing the infiltration abundance of 28 immune cells between subtypes (Figure 5G), and the results showed that 25 of these immune cells were significantly different between groups.

### 3.6. Establishment and Validation of a Prognostic Nomogram Model

To study the prognostic value of key genes, we analyzed the clinical information of patients from the TCGA-PRAD dataset (Table S5). Univariable Cox regression analysis was performed on the key genes, and variables with *p*-value < 0.1 were included in the multivariable Cox regression analysis to construct a prognostic model (Figure S5A) and develop a nomogram (Figure S5B). The genes included in the nomogram model were defined as prognostic key genes. Calibration curves (Figure S5C–E) and DCA curves (Figure S5F–H) demonstrated good predictive performance in predicting progression-free survival at 1-year, 3-year, and 5-year time points.

Patients were then stratified into low-risk and high-risk groups based on the median risk score from the nomogram model. Kaplan-Meier curves (Figure S6A) and ROC curves (Figure S6B) confirmed the good discriminative ability and predictive efficacy of the constructed model. Additionally, significant differences were observed between high-risk and low-risk patients in terms of MSI (Figure S6C), TMB (Figure S6D), Stromal-score (Figure S6E), Immune-score (Figure S6F), ESTIMATE-score (Figure S6G), and Tumor Purity-score (Figure S6H). Specifically, risk-score was negatively correlated with StromalScore (R = −0.35, *p* = 2.9 × 10^−16^, Figure S6I), ImmuneScore (R = −0.15, *p* = 0.00067, Figure S6J), and ESTIMATEScore (R = −0.26, *p* = 3.7 × 10^−9^, Figure S6K), while positively correlated with Tumor Purity (R = 0.26, *p* = 3.7 × 10^−9^, Figure S6L).

### 3.7. Upregulation of CAV1, PALLD, and ITGB8 and Downregulation of CLDN7 in Prostate Cancer Cell Lines

**Figure 6:**
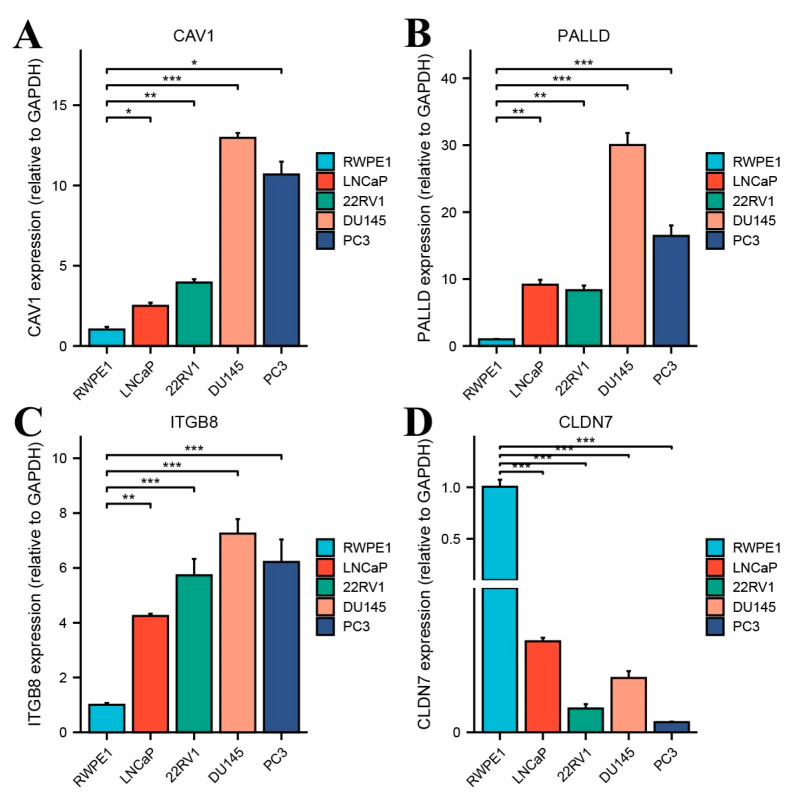
Differential expression of CAV1, PALLD, ITGB8, and CLDN7 in prostate cancer cell lines and normal prostate epithelial cells. RT-qPCR was used to measure the relative mRNA expression levels of four genes: CAV1 (**A**), PALLD (**B**), ITGB8 (**C**), and CLDN7 (**D**) in the normal prostate epithelial cell line RWPE1 and four prostate cancer cell lines (LNCaP, 22RV1, DU145, and PC3). GAPDH was used as an internal control for normalization. * *p* < 0.05, ** *p* < 0.01, *** *p* < 0.001.

Four prognostic key genes were identified in the in vitro experiments, which were consistent with the bioinformatics analysis. The expression levels of CAV1, PALLD, ITGB8, and CLDN7 were analyzed using RT-qPCR in the normal prostate epithelial cell line RWPE1 and four prostate cancer cell lines: LNCaP, 22RV1, DU145, and PC3. The results revealed that CAV1 (Figure 6A) was significantly upregulated in all PCa cell lines compared to RWPE1, with the highest expression observed in DU145 and PC3 cells, which was significantly higher than that in LNCaP and RWPE1 cells (*p* < 0.05, ** *p* < 0.001). PALLD (Figure 6B) expression was markedly elevated in DU145 cells, showing a significant increase compared to RWPE1, LNCaP, and 22RV1 cells (** *p* < 0.001). PC3 cells also displayed elevated PALLD expression, although to a lesser extent, while LNCaP and 22RV1 showed relatively low PALLD levels. For ITGB8 (Figure 6C), its expression was significantly increased in PCa cell lines relative to RWPE1 (* *p* < 0.01, ** *p* < 0.001), with DU145 and PC3 showing the highest levels and LNCaP and 22RV1 displaying moderate expression. Conversely, CLDN7 (Figure 6D) was significantly downregulated in all PCa cell lines compared to RWPE1 (** *p* < 0.001). RWPE1 exhibited the highest CLDN7 expression, while LNCaP, 22RV1, DU145, and PC3 cells displayed markedly reduced levels. These findings suggest that CAV1, PALLD, and ITGB8 are upregulated, while CLDN7 is downregulated in prostate cancer cell lines, highlighting their potential roles in prostate cancer progression.

### 3.8. The Functional Role of PALLD

**Figure 7:**
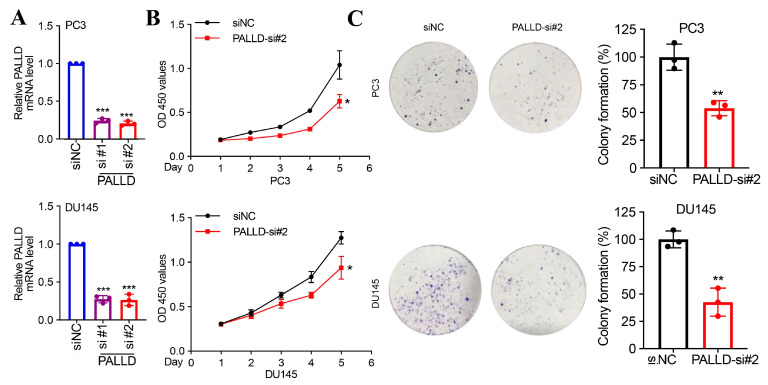
(**A**) RT-qPCR analysis shows significant knockdown of PALLD expression by siRNAs (si#1, si#2) in PC3 and DU145 cells compared to the control (siNC); (**B**) CCK-8 assays indicate reduced proliferation in PC3 and DU145 cells after PALLD knockdown (PALLD-si#2); (**C**) Colony formation assays show a significant decrease in colony formation in PC3 and DU145 cells following PALLD knockdown (PALLD-si#2). * *p* < 0.05, ** *p* < 0.01, *** *p* < 0.001.

Since PALLD has not been previously reported in prostate cancer, we further tested its function in vitro. We investigated the functional role of the PALLD gene in PC3 and DU145 cell lines. First, we observed a significant reduction in PALLD mRNA levels in cells treated with siRNA targeting PALLD (si#1 and si#2) compared to non-targeting control (siNC) in both PC3 (Figure 7A, top left) and DU145 (Figure 7A, bottom left) cells. The differences were highly statistically significant (*p* < 0.001).

Next, cell proliferation assays indicated a significant reduction in proliferation rates in PALLD-si#2 treated cells compared to siNC-treated cells over five days for both PC3 (Figure 7B, top middle) and DU145 (Figure 7B, bottom middle) cell lines (*p* < 0.05). Specifically, from day 2 onwards, the proliferation rate of PALLD-si#2 treated cells was significantly lower than that of the siNC group, with notable differences by day 5.

Additionally, colony formation assays demonstrated a substantial decrease in colony formation ability in PALLD-si#2 treated PC3 (Figure 7C, top right) and DU145 (Figure 7C, bottom right) cells (*p* < 0.01). Microscopic observations showed fewer colonies in the PALLD-si#2 group compared to the control group.

These results collectively suggest that PALLD plays a crucial role in maintaining the proliferation and colony-forming ability of PC3 and DU145 cancer cell lines, with its knockdown significantly inhibiting these cellular functions.

### 3.9. CIBERSORT Analysis

In the TCGA-PRAD dataset, we utilized the CIBERSORT algorithm to estimate the infiltration levels of 22 immune cell types in high-risk and low-risk group samples. Inter-group differences in infiltration levels were then assessed using the Mann-Whitney U test (Figure S7A). Statistically significant differences were observed in the infiltration levels of nine immune cell types: naive B cells, CD8 T cells, resting CD4 memory T cells, follicular helper T cells, regulatory T cells (Tregs), resting NK cells, activated NK cells, resting dendritic cells, and neutrophils. We also examined the correlations among the infiltration levels of these nine immune cell types, presenting the results. Negative correlations were found among these immune cell types in both the low-risk (Figure S7B) and high-risk groups (Figure S7C).

Moreover, we analyzed the correlations between the infiltration levels of these nine immune cell types and the expression levels of key prognostic genes. Results with *p*-values < 0.05 were filtered and visualized using correlation dot plots (Figure S7D,E). The analysis identified significant positive correlations between the infiltration levels of naive B cells and resting CD4 memory T cells and the expression levels of the prognostic key genes.

### 3.10. Drug Sensitivity Analysis

We used drug sensitivity data from the GDSC database as a training set to assess the sensitivity of PARD to common anticancer drugs. We selected the top 20 drugs with significant inter-group differences for presentation (Figure S8). Our findings indicated a general trend of higher drug sensitivity in the high-risk group compared to the low-risk group. These results underscore the importance of personalized treatment for PARD patients.

## 4. Discussion

Mitophagy could maintain the integrity of the mitochondrial pool by selectively targeting the elimination of damaged, dysfunctional or senescent mitochondria through lysosomal degradation [16,17]. In addition, mitophagy allows cells to survive under stressful conditions such as hypoxia by reducing mitochondrial mass, decreasing reactive oxygen species production, and reducing nutrient consumption [18]. Mitophagy is also involved in programmed events during development and differentiation, such as the elimination of parental mitochondria from fertilized eggs and the removal of mitochondria during erythropoiesis and muscle differentiation [19]. Mitophagy also plays an important role in tumorigenesis, progression and therapy resistance, and some proteins responsible for mitophagy have been found to be dysregulated in malignant tumors [20,21]. Notably, mitophagy has also been found to interact with TLR9 to promote the production of the chemokine CXCL10 by cancer cells, which further promotes T-cell recruitment and thus enhances the efficacy of immunotherapy [22]. MRGs have been shown to assist in patient risk stratification, identify patient subgroups that may potentially benefit from immunotherapy, and optimize the implementation of precision medicine in a variety of cancers, including gastric, lung, and prostate cancer [23,24,25]. Thus, for patients with refractory advanced prostate cancer who have failed androgen deprivation therapy, androgen receptor signaling inhibitors, and docetaxel, the exploration of new targets against MRGs could provide new perspectives on the underlying mechanisms of prostate cancer and ultimately contribute to a broader understanding of this complex disease [26,27,28].

Through screening of the bulk transcriptome, we confirmed that 26 key genes associated with mitophagy (CAV1, MARCKSL1, TGFBR3, PALLD, GPM6B, PDLIM1, ANXA2, ACSL4, EHD2, TACC1, SERPINB1, ITGB8, STOM, EPS8, PHLDA1, PDLIM5, LGALS3BP, HSPB1, EPAS1, CXCL12, KRT18, LMCD1, CSRP2, BST2, CPVL, CLDN-7) were significantly enriched in the hypoxia, response to decreased oxygen levels, response to oxygen levels-related BP pathway. Using key genes, it was possible to categorize PARD patients into two distinct subtypes, with the vast majority of key genes in cluster1 significantly upregulated relative to cluster2 between the two subtypes. Using the ssGSEA algorithm to explore the infiltration abundance of immune cells between the different subtypes also demonstrated that the vast majority of immune cells were significantly different between groups. The key genes associated with prognosis were further screened by COX regression analysis, and the prediction model constructed by seven prognostic key genes (Cav1, CLDN-7, ITGB8, PALLD, PDLIM5, TACC1, and TGFBR3) accurately predicted the prognosis of PARD and differentiated between high- and low-risk patients.

PALLD is overexpressed in strongly aggressive cancer cells, so dysregulation of PALLD may promote invasive/invasive pathological behavior of cancer cells [29]. Our findings provide strong evidence that PALLD plays a critical role in promoting the proliferation and colony-forming ability of prostate cancer cells. Knockdown of PALLD expression in PC3 and DU145 cell lines led to a significant reduction in both cell proliferation and colony formation, indicating that PALLD is essential for maintaining the growth and survival of these cancer cells. Specifically, our proliferation assays demonstrated that silencing PALLD resulted in a marked decrease in proliferation rates starting from day 2, with more pronounced effects by day 5. Similarly, colony formation assays revealed a substantial reduction in colony formation ability, which was consistent across both cell lines. These results suggest that PALLD is a key regulator of cell proliferation and tumorigenic potential in prostate cancer, highlighting its potential as a therapeutic target in the treatment of prostate cancer. Furthermore, these findings align with previous studies implicating cytoskeletal regulators, such as PALLD, in cancer progression and metastasis, reinforcing the idea that modulating the actin cytoskeleton may offer a promising strategy for cancer therapy. As a mitochondria-associated gene, PALLD has not been previously studied in prostate cancer. For the first time, we demonstrated its pro-oncogenic function through in vitro cellular experiments.

Lastly, drug sensitivity data from the GDSC database were used as a training set to predict PRAD sensitivity to common anticancer drugs, of which 20 drugs differed significantly between the high and low-risk groups and were generally shown to be drug sensitivity in the high-risk group was higher than that in the low-risk group. Using a genome-wide lentiviral small hairpin RNA library screen, the PDPK1 inhibitor BX-795 has been shown to significantly reduce tumor-specific cell growth and synergize with docetaxel to enhance treatment sensitivity in prostate cancer cells [30]. The γ secretase inhibitors BMS-708163 were found to eliminate enzalutamide resistance in vitro by inhibiting Notch1 or/and Notch4 [31]. Embelin, an active component of the traditional herb Embelia ribes, has been found to downregulate AR expression and decrease androgen-mediated AR phosphorylation at Ser [32], induce apoptosis in prostate cancer cells by modulating the Akt and β-catenin signaling pathways [33], as well as inhibit the growth of prostate tumors in conjunction with bicalutamide [34], and even enhance the therapeutic effects of the treatment in collaboration with radiation therapy [35]. These findings provide new insights into the relationship between mitophagy and systemic therapeutic strategies for prostate cancer, further emphasizing the importance of individualized treatment for cancer patients.

The current study has some limitations. First, the inevitable selection bias of retrospective studies and the limited clinicopathologic information from database mining could result in studies that do not provide efficient and complete results. Second, there is still room for improvement in the performance of the constructed model, and more biomarkers should be included to improve the predictive ability of the model. Finally, experimental immunohistochemical corroboration is needed, as well as more ex vivo and in vivo experiments to validate our conclusions.

## 5. Conclusions

This study identified MRGs significantly associated with prognosis that could be used as biomarkers for the management of patients with PARD and developed a score prediction model. In addition, MRGs could distinguish patients with PARD by unsupervised clustering, and there are significant differences in responses to immunotherapy and other treatments between different subtypes. This study provides new insights into the relationship between mitophagy and PARD and contributes to future research in this field; however, further studies are needed to validate specific disease mechanisms and molecular targets and bridge the gap between research findings and clinical application.

## Data Availability

Data supporting the findings of this study are available from the corresponding author upon reasonable request.

## Supplementary Materials

The following supporting information can be downloaded at: https://www.mdpi.com/article/10.3390/biomedicines13020311/s1, Table S1: Prostate cancer sequencing dataset information list; Table S2: Mitophagy-related genes list; Table S3: The primer sequence of seven prognostic key genes; Table S4: The 160 mitophagy-related single-cell differently expressed genes list; Table S5: Characteristics of patients with prostate cancer. Characteristics of patients with prostate cancer; Figure S1: The workflow of the study; Figure S2: (A) Quality control of scRNA-seq data; (B) The variance plot showed 21,191 genes in all cells, with red dots representing the top 3000 highly variable genes; (C) 27 clusters were visualized based on the uniform manifold approximation and projection algorithm; Figure S3: (A–C) Differentiation trajectory of fibroblasts, colored for types (A), pseudotime (B), and state (C); (D) Trajectory of six fibroblasts subtypes; (E) Heatmap of differently expressed genes of six fibroblasts subtypes; Figure S4: (A) Gene Ontology enrichment analysis of key genes; (B–D) Gene Ontology enrichment analysis in biological process (B), cellular component (C), molecular function (D); Figure S5: (A) Forest plot of univariate Cox regression result for seven prognostic key genes; (B) The construction of the nomogram; (C–E) The calibration curve for assess the agreement at 1-(C), 3-(D) and 5-(E) year PFS, respectively; (F–H) The decision curve analysis curves compared for 1-(F), 3-(G) and 5-(H) year PFS, respectively; Figure S6: (A) The Kaplan-Meier curves for the high and low-risk groups; (B) The area under the curve of the nomogram compared for 1-, 3-, and 5-year PFS; (C–H) MSI (C), TMB (D), Stromal-score (E), Immune-score (F), ESTIMATE-score (G), and Tumor Purity-score (H) in the high and low-risk groups, respectively; (I–L) Correlation between StromalScore (I), ImmuneScore (J), ESTIMATEScore (K), Tumor Purityscore (L) and riskscore; Figure S7: (A) The box plot illustrates the immune infiltration profiles of the high and low-risk groups assessed using CIBERSORT; (B,C) Correlation analysis of immune cell infiltration abundance in the low (B) and high (C) risk groups; (D,E) Correlation analysis of immune cell and seven prognostic key genes in the low (D) and high (E) risk groups; Figure S8: Evaluation of drug sensitivity. The comparisons in IC50 value of AZD6482 (A), CCT007093 (B), BX.795 (C), DMOG (D), VX.702 (E), BMS.708163 (F), AP.24534 (G), Embelin (H), Imatinib (I), AICAR (J), AZD.0530 (K), Bexarotene (L), KIN001.135 (M), Lapatinib (N), Z.LLNle.CHO (O), PAC.1 (P), AS601245 (Q), CGP.082996 (R), JNK.Inhibitor.VIII (S), and PF.562271 (T) in the high and low-risk groups.

## Abbreviations

PARD, prostate adenocarcinoma; TME, tumor microenvironment; scRNA-seq, single-cell RNA sequencing; MRGs, mitophagy-related genes; GEO, Gene Expression Omnibus; TCGA, the Cancer Genome Atlas; MSI, microsatellite instability; TMB, tumor mutational burden; PCA, principal component analysis; PCs, principal components; UMAP, uniform manifold approximation and projection; GO, gene ontology; CDF, cumulative distribution function; ROC, receiver operating characteristic; DCA, decision curve analysis; IC50, half-maximal inhibitory concentration; GDSC, Genomics of Drug Sensitivity in Cancer; MR-scDEGs, mitophagy-related single-cell differently expressed genes; CC, cell component; MF, molecular function.
