# Correction: Zhang et al. An Integrated Approach Utilizing Single-Cell and Bulk RNA-Sequencing for the Identification of a Mitophagy-Associated Genes Signature: Implications for Prognostication and Therapeutic Stratification in Prostate Cancer. *Biomedicines* 2025, *13,* 311

**DOI:** 10.3390/biomedicines13102501

**Published:** 2025-10-14

**Authors:** Yuke Zhang, Li Ding, Zhijin Zhang, Liliang Shen, Yadong Guo, Wentao Zhang, Yang Yu, Zhuoran Gu, Ji Liu, Aimaitiaji Kadier, Jiang Geng, Shiyu Mao, Xudong Yao

**Affiliations:** 1 Department of Urology, Shanghai Tenth People’s Hospital, Tongji University School of Medicine, 301 Middle Yan Chang Road, Shanghai 200072, China; yukezhang001@gmail.com (Y.Z.); ding0710@foxmail.com (L.D.); 2231200@tongji.edu.cn (Z.Z.); 1731191@tongji.edu.cn (Y.G.); zhangwentao98@163.com (W.Z.); sdykdxyy@163.com (Y.Y.); guzhuorango@126.com (Z.G.); drliu7087797@163.com (J.L.); 1911637@tongji.edu.cn (A.K.); gengjiangsn@sina.com (J.G.); 2 Urologic Cancer Institute, Tongji University School of Medicine, Shanghai 200072, China; 3 Department of Urology, Ningbo Yinzhou People’s Hospital, 251 Baizhang East Road, Ningbo 315100, China; shenliliang@sina.com

**References**

The original reference 12 has been retracted and its contents are no longer of reference value. The authors would like to make the following corrections for their published paper [1]:

Deleting original reference 12:

12. Yan, H.; Xiao, F.; Zou, J.; Qiu, C.; Sun, W.; Gu, M.; Zhang, L. NR4A1-induced increase in the sensitivity of a human gastric cancer line to TNFα-mediated apoptosis is associated with the inhibition of JNK/Parkin-dependent mitophagy. *Int. J. Oncol.* **2018**, *52*, 367–378.

With this correction, the order of some references has been adjusted accordingly. The authors state that the scientific conclusions are unaffected. This correction was approved by the Academic Editor. The original publication has also been updated.
