# Supplementary material for: An Integrated Approach Utilizing Single-Cell and Bulk RNA-Sequencing for the Identification of a Mitophagy-Associated Genes Signature: Implications for Prognostication and Therapeutic Stratification in Prostate Cancer
Source: Biomedicines. 2025 Jan 27;13(2):311. doi: 10.3390/biomedicines13020311 (PMC11853322; doi:10.3390/biomedicines13020311)
Supplement: Supplementary file 1 [file biomedicines-13-00311-s001.zip › Supplement Tables S1 S3 S4 S5.pdf]

Table S1. Prostate cancer sequencing dataset information list					
	TCGA-PRAD	GSE185344	GSE46602	GSE69223	GSE38241
Platform	TCGA	GPL24676	GPL570	GPL570	GPL4133
Species	Homo sapiens	Homo sapiens	Homo sapiens	Homo sapiens	Homo sapiens
Experiment type	/	Expression profiling by high throughput sequencing	Expression profiling by array	Expression profiling by array	Expression profiling by array
Tissue	Prostate tissues	Prostate tissues	Prostate tissues	Prostate tissues	Prostate tissues
Samples in PRAD/Tumor group	499	7	36	15	18
Samples in Control/Benign group	52	7	14	15	21
Reference	/	Single cell analysis of cribriform prostate cancer reveals cell intrinsic and tumor microenvironmental pathways of aggressive disease.	Expression profiling of prostate cancer tissue delineates genes associated with recurrence after prostatectomy.	Integration of tissue metabolomics, transcriptomics and immunohistochemistry reveals ERG- and gleason score-specific metabolomic alterations in prostate cancer.	DNA methylation alterations exhibit intraindividual stability and interindividual heterogeneity in prostate cancer metastases.
TCGA，The cancer genome atlas；PRAD，Prostate Cancer					

Table S3. The primer sequence of seven prognostic key genes		
Gene	Primers	Sequence
GAPDH	Forward	AGCCCAAGATGCCCTTCAGT
GAPDH	Reverse	CCGTGTTCCTACCCCCAATG
CAV1	Forward	GCGACCCTAAACACCTCAAC
CAV1	Reverse	ATGCCGTCAAAACTGTGTGTC

CLDN7	Forward	AGCTGCAAAATGTACGACTCG
CLDN7	Reverse	GGAGACCACCATTAGGGCTC
ITGB8	Forward	ACCAGGAGAAGTGTCTATCCAG
ITGB8	Reverse	CCAAGACGAAAGTCACGGGA
PALLD	Forward	AAGAAGGCCAGTAGAACTGCT
PALLD	Reverse	AAGCGAAGTTTTCGTTCCAGG
PDLIM5	Forward	AAGAATAGGCGATGTGGTTCTCA
PDLIM5	Reverse	GCAGCAGATGCTCTTTGCAG
TACC1	Forward	AGGGGCAGTGATCTCCCAG
TACC1	Reverse	TTTCTGACCACATGACGTGGA
TGFBR3	Forward	TGGGGTCTCCAGACTGTTTTT
TGFBR3	Reverse	CTGCTCCATACTCTTTTCGGG

Table S4. 160 mitophagy-related single-cell differently expressed genes list					
A2M	CLU	GLUL	KRT19	NFKB1	SELENOK
ACSL4	CNN3	GNLY	KRT8	NME4	SERPINA1
ACTA2	COL15A1	GPM6B	LGALS1	NPC2	SERPINB1
ACTB	COL1A1	GRN	LGALS3BP	NR4A1	SFN
AKAP13	CPA3	GSN	LMCD1	NUPR1	SLC18A2
ANGPTL7	CPVL	GZMH	LMNA	P4HB	SLC2A3
ANXA1	CRTAP	HLA-A	MAOB	PALLD	SOD2
ANXA2	CSRP1	HLA-B	MAPK1	PDK4	SORD
APOE	CSRP2	HLA-C	MARCKSL1	PDLIM1	SPTBN1
APP	CTSD	HMOX1	MCTP1	PDLIM5	SQSTM1
ARPC1B	CXCL12	HSP90AA1	MDK	PFN1	SRP14
AZGP1	CYB5R3	HSPA1A	MFGE8	PHLDA1	STOM
BAG3	DCN	HSPA1B	MIF	PLP1	TACC1
BST2	DHRS7	HSPA6	MMP2	PNP	TAGLN2
CACYBP	DNAJB1	HSPA8	MNDA	PRCP	TGFBR3
CALD1	DSTN	HSPB1	MT1M	RAC2	THBS1
CALM2	EHD2	HSPD1	MT-CO2	RCAN1	TMSB4X
CAV1	EHD4	HSPG2	MT-CO3	RDX	TPM1
CCL2	EPAS1	HSPH1	MT-ND1	REL	TUBA1A
CCN1	EPS8	IFI16	MT-ND4	RGS1	TUBA4A
CCN2	FABP5	IL1B	MT-ND5	RGS13	TUBB2B
CD3D	FHL1	IL33	MYC	S100A8	TXNIP
CD44	FLNA	ISYNA1	MYH11	S100A9	VIM
CD9	FLT1	ITGA6	MYLK	S100B	YWHAH
CD93	FOS	ITGB1	NDRG2	SARAF	YWHAZ
CKB	FTH1	ITGB8	NDUFA4L2	SDCBP	ZG16B
CLDN7	GALC	KRT18	NEDD4L		

Table S5. Characteristics of patients with prostate cancer	
Characteristics	Overall
Pathologic T stage, n (%)	
T2	189 (38.3%)
T3	294 (59.5%)
T4	11 (2.2%)
Pathologic N stage, n (%)	
N0	348 (81.3%)
N1	80 (18.7%)
Clinical T stage, n (%)	
T1	177 (43.5%)
T2	175 (43%)
T3	53 (13%)
T4	2 (0.5%)
Clinical M stage, n (%)	
M0	457 (99.3%)
M1	3 (0.7%)
Age, n (%)	
<= 60	225 (44.9%)
> 60	276 (55.1%)
OS event, n (%)	
Alive	491 (98%)
Dead	10 (2%)
DSS event, n (%)	
No	494 (99%)
Yes	5 (1%)
PFI event, n (%)	
No	407 (81.2%)
Yes	94 (18.8%)
