# Supplementary figures and images for: An Integrated Approach Utilizing Single-Cell and Bulk RNA-Sequencing for the Identification of a Mitophagy-Associated Genes Signature: Implications for Prognostication and Therapeutic Stratification in Prostate Cancer
Source: Biomedicines. 2025 Jan 27;13(2):311. doi: 10.3390/biomedicines13020311 (PMC11853322; doi:10.3390/biomedicines13020311)

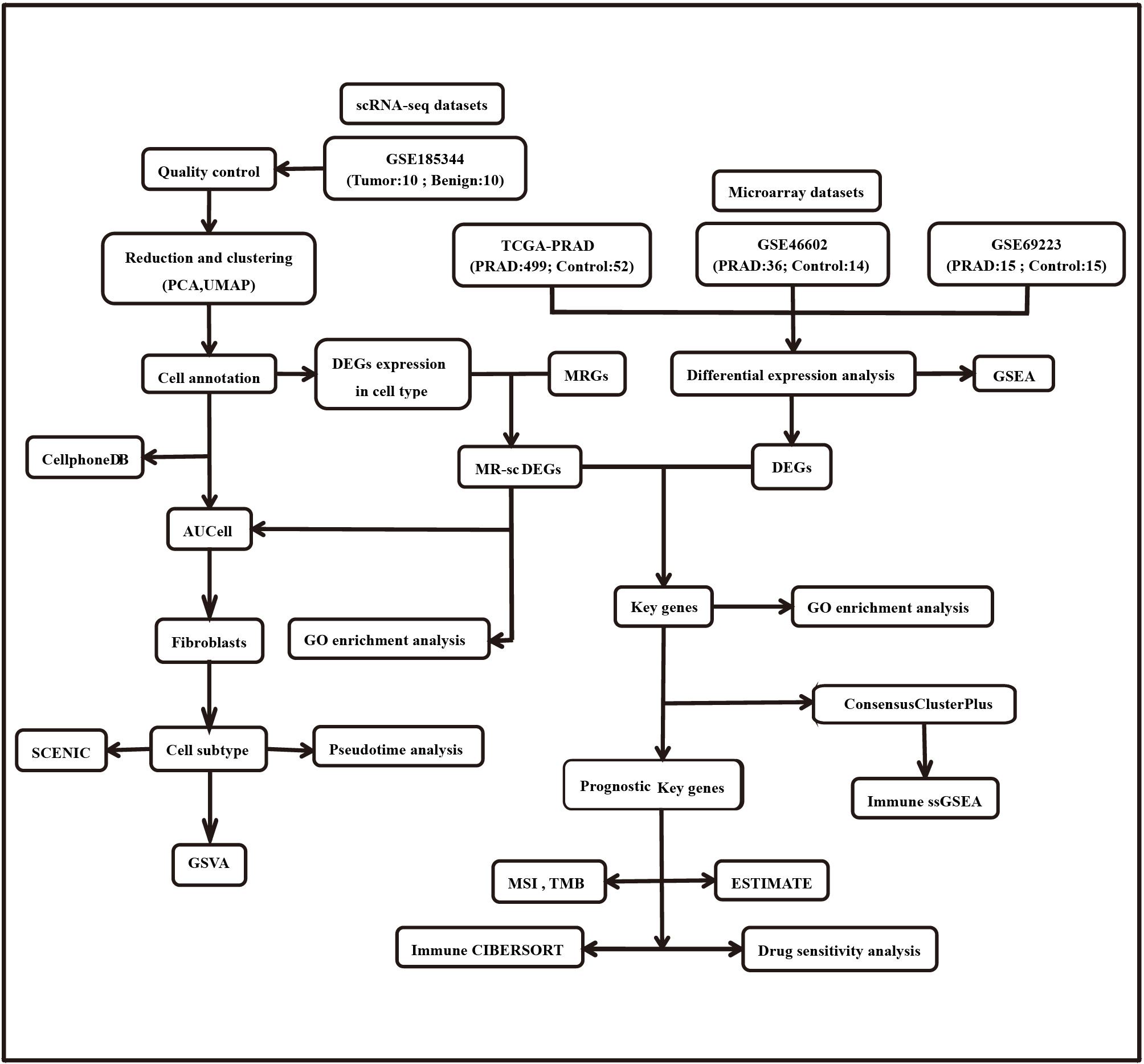

Supplement: Supplementary file 1 [file biomedicines-13-00311-s001.zip › FigureS1.jpg]

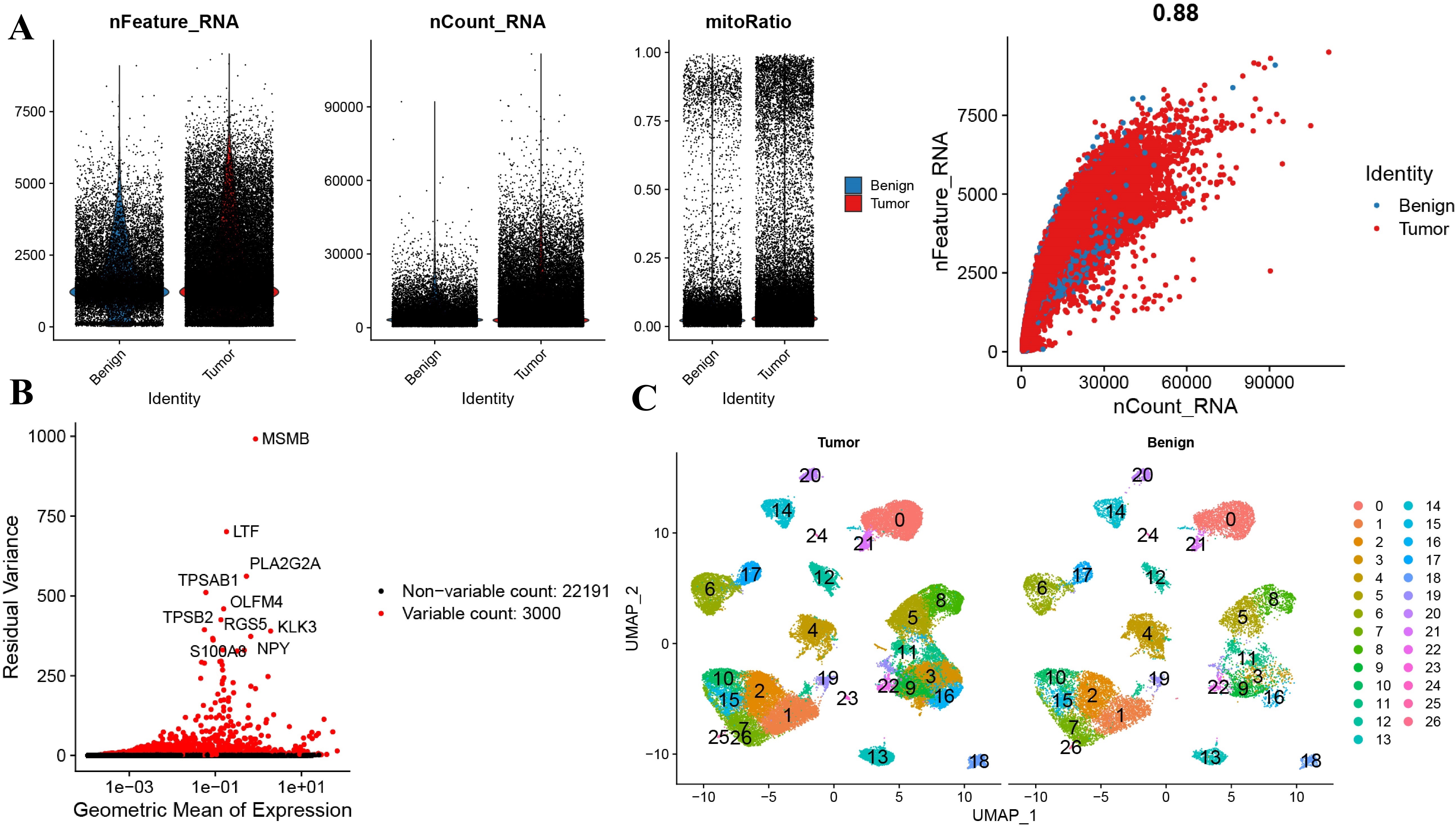

Supplement: Supplementary file 1 [file biomedicines-13-00311-s001.zip › FigureS2.jpg]

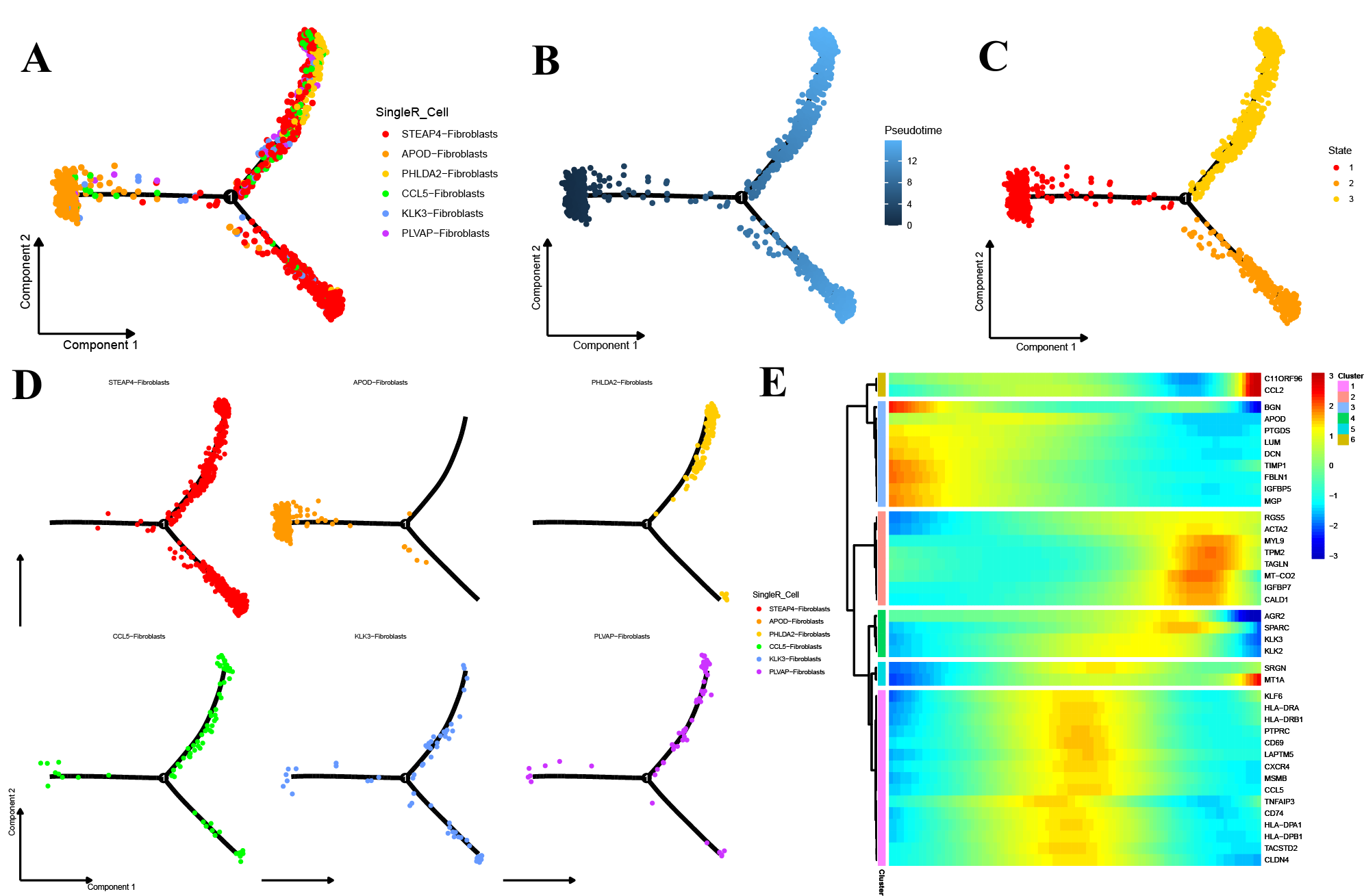

Supplement: Supplementary file 1 [file biomedicines-13-00311-s001.zip › FigureS3.tif]

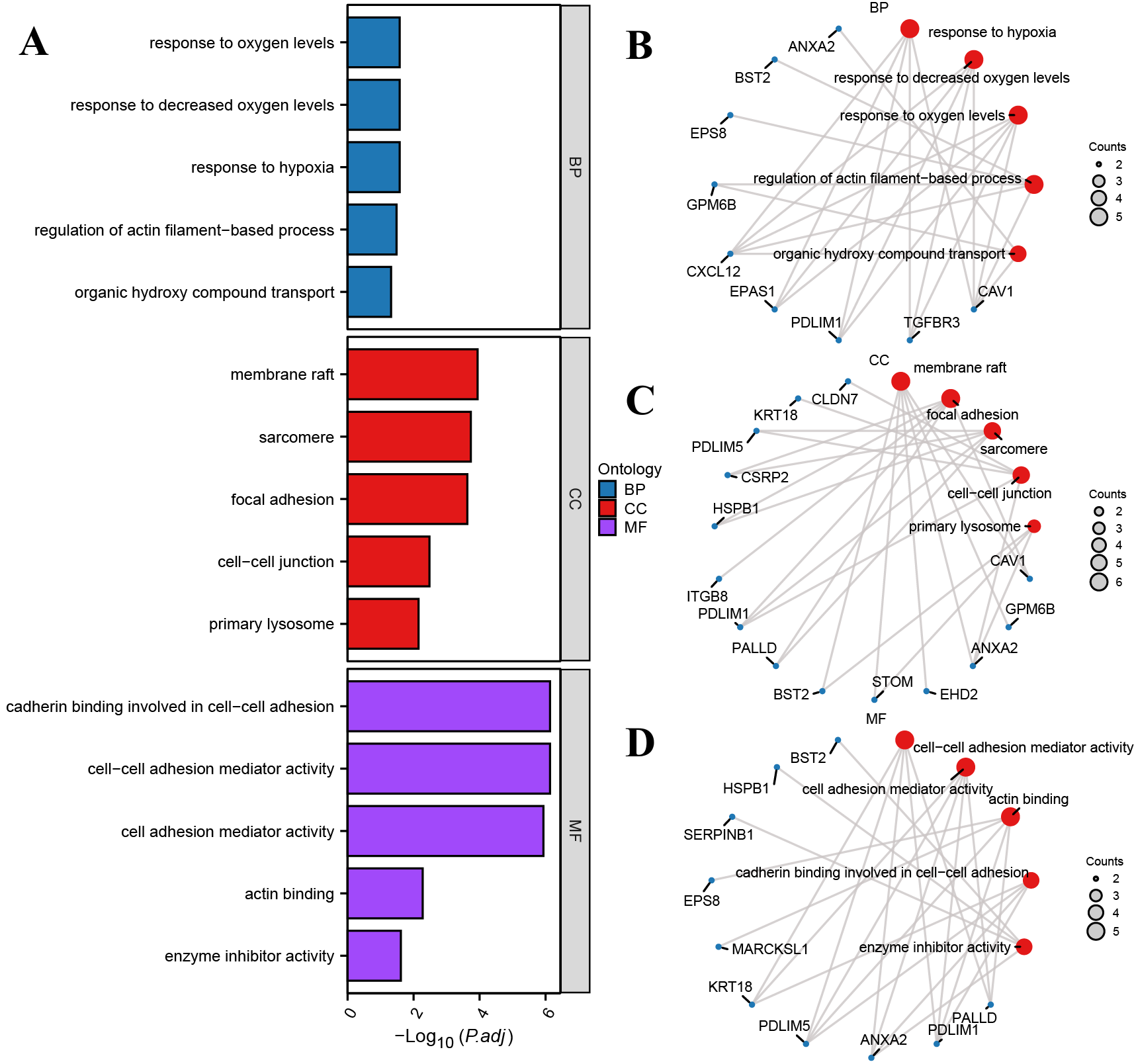

Supplement: Supplementary file 1 [file biomedicines-13-00311-s001.zip › FigureS4.tif]

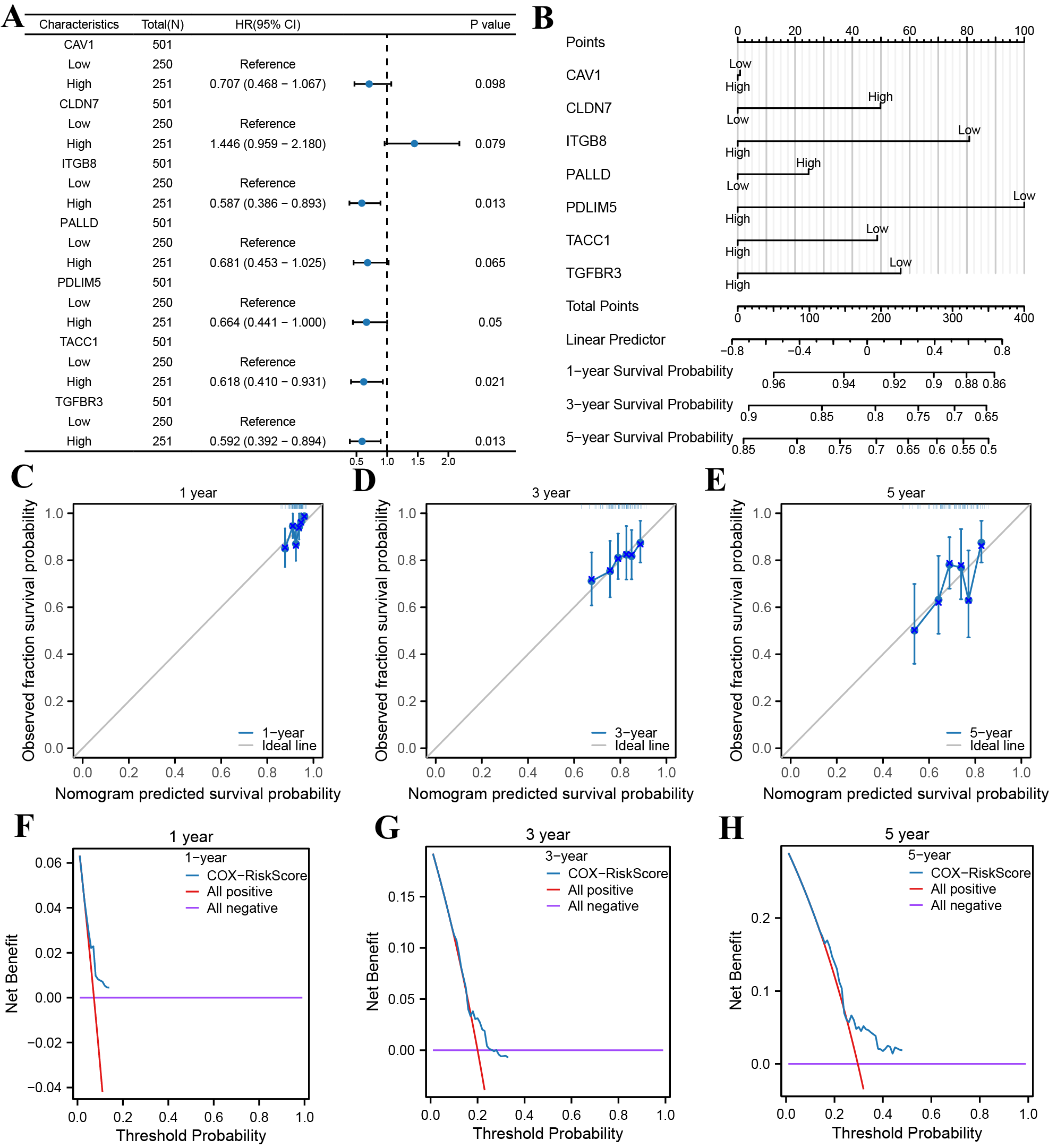

Supplement: Supplementary file 1 [file biomedicines-13-00311-s001.zip › FigureS5.tif]

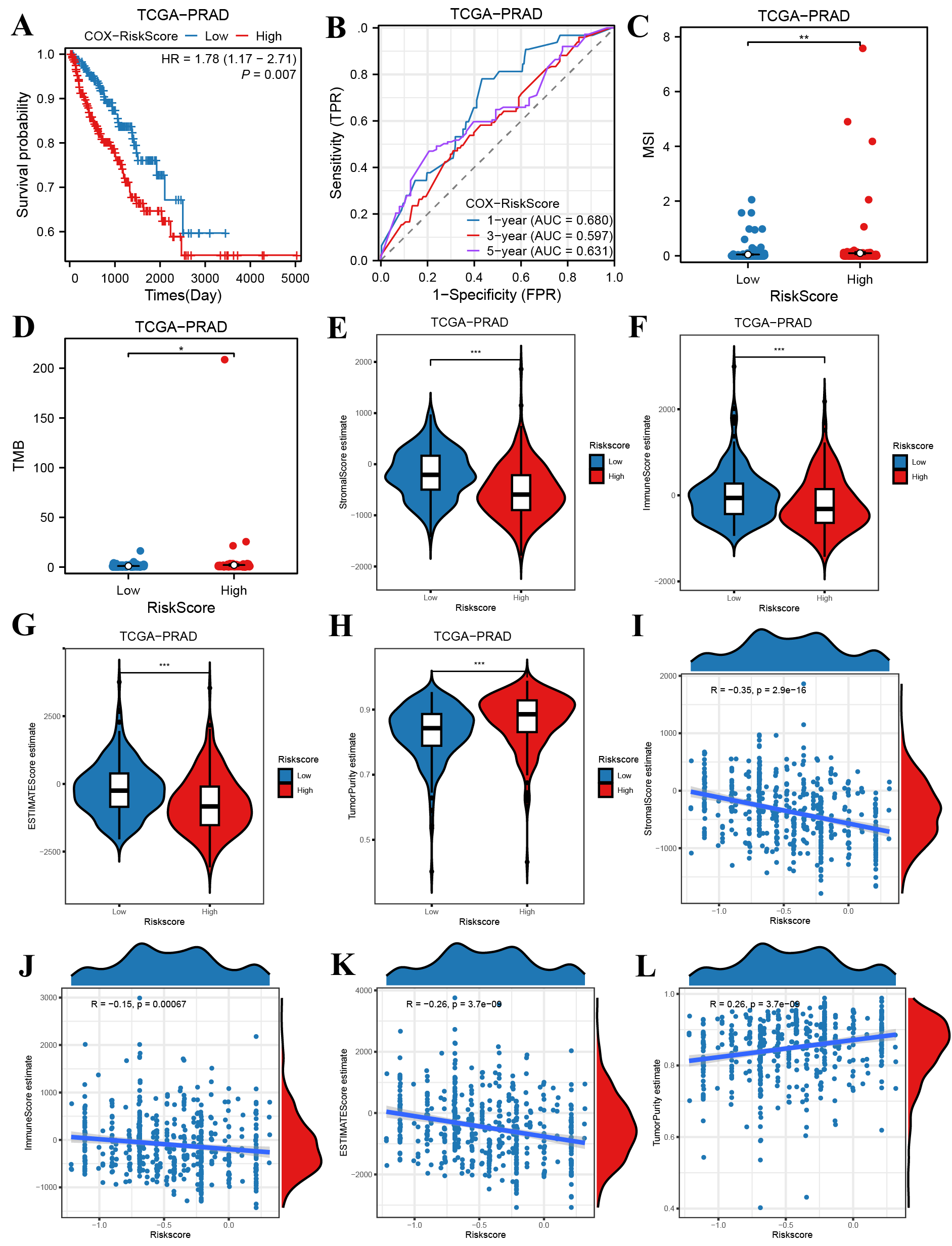

Supplement: Supplementary file 1 [file biomedicines-13-00311-s001.zip › FigureS6.tif]

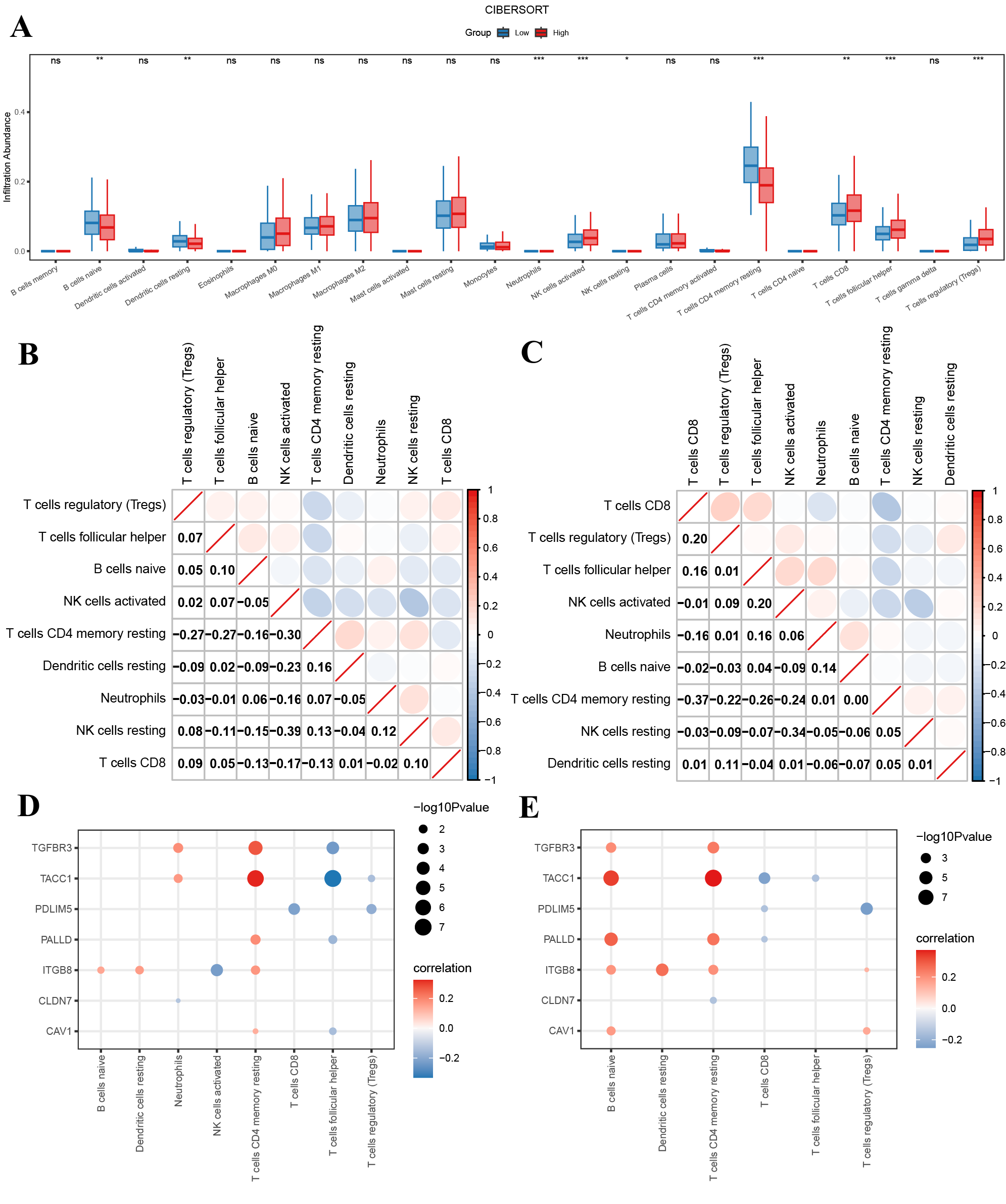

Supplement: Supplementary file 1 [file biomedicines-13-00311-s001.zip › FigureS7.tif]

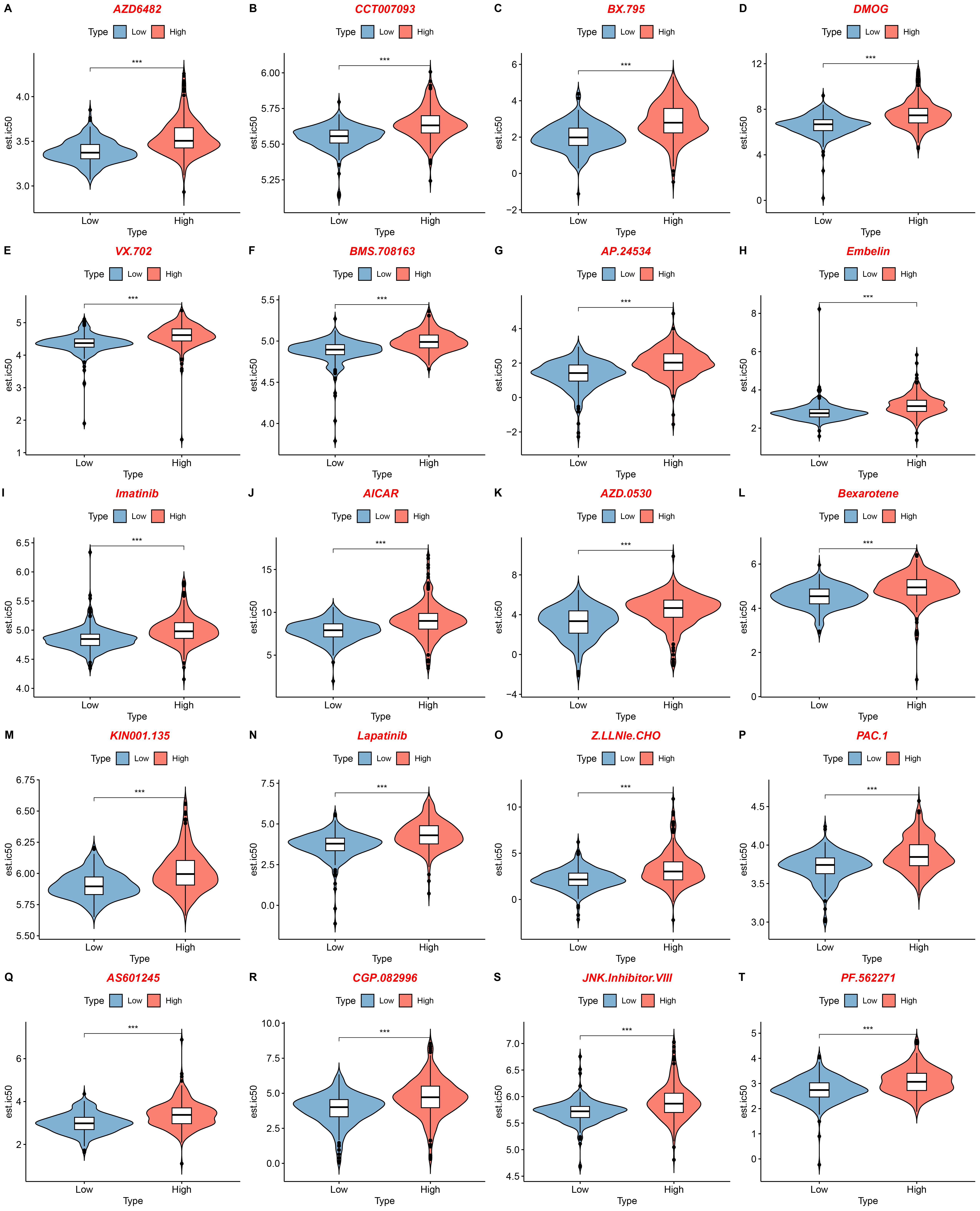

Supplement: Supplementary file 1 [file biomedicines-13-00311-s001.zip › FigureS8.tif]
